# Cochlear Organ Dissection, Immunostaining, and Confocal Imaging in Mice

**DOI:** 10.21769/BioProtoc.5167

**Published:** 2025-01-20

**Authors:** Chenyu Chen, Binjun Chen, Xiaoqing Qian, Haojie Sun, Xiao Fu, Dongdong Ren

**Affiliations:** 1 ENT Institute and Department of Otorhinolaryngology, Eye & ENT Hospital, Fudan University, Shanghai, China; 2 NHC Key Laboratory of Hearing Medicine Research, Eye & ENT Hospital, Fudan University, Shanghai, China; 3 Department of Otorhinolaryngology, Shanghai Ninth People's Hospital, Shanghai Jiao Tong University School of Medicine, Shanghai, China

**Keywords:** Cochlea, Hair cells, Dissection, Immunostaining, Confocal Imaging

## Abstract

The organ of Corti, located in the inner ear, is the primary organ responsible for animal hearing. Each hair cell has a V-shaped or U-shaped hair bundle composed of actin-filled stereocilia and a kinocilium supported by true transport microtubules. Damage to these structures due to noise exposure, drug toxicity, aging, or environmental factors can lead to hearing loss and other disorders. The challenge when examining auditory organs is their location within the bony labyrinth and their small and fragile nature. This protocol describes the dissection procedure for the cochlear organ, followed by confocal imaging of immunostained endogenous and fluorescent proteins. This approach can be used to understand hair cell physiology and the molecular mechanisms required for normal hearing.

Key features

• Protocol for the microdissection of the organ of Corti and suitable preparation for later immunostaining.

• This technique involves the evaluation of mouse cochlea for planar-cell-polarity protein.

• Quantitative and qualitative analysis of hair cell cilia in different dimensions.

## Graphical overview


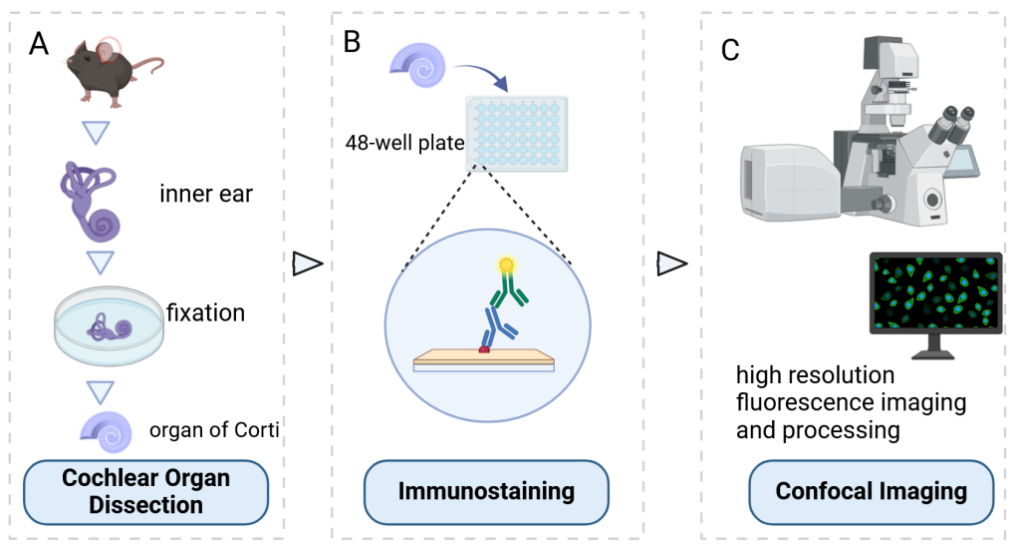


## Background

**Figure 1.**
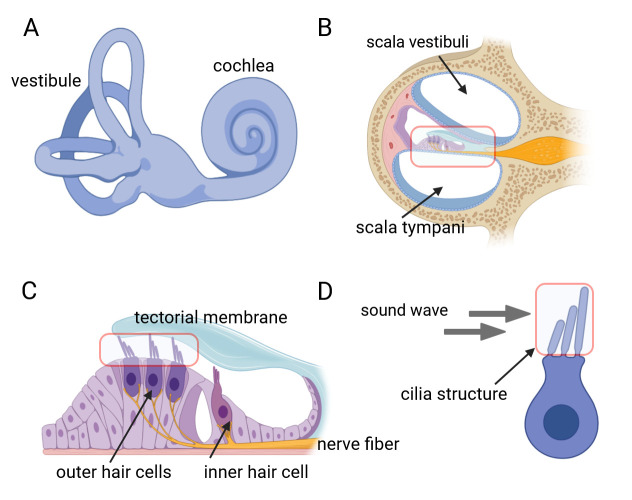
Inner ear anatomy. A, B) The cochlea and a close-up of a single turn cross-section, revealing the organ of Corti. C) The organ of Corti contains mechanosensitive hair cells, which lie beneath the tectorial membrane. D) Hair bundles composed of actin-filled stereocilia arranged in a staircase configuration.

The inner ear, serving as both the auditory organ and local sensory receptor, houses the critical auditory sensor [1,2]. It consists of three rows of outer hair cells and one row of inner hair cells, as well as various supporting cells. These cellular components are meticulously arrayed in a mosaic pattern on the intricate cochlear membrane of the inner ear, demonstrating remarkable flexibility [3–6]. Hair cells convert acoustic signals into electrical signals through the deflection of tip cilia. These cilia are important cellular structures involved in normal morphogenesis and functional activities of hair cells [7,8]. Electrical impulses are ultimately channeled to the cerebral cortex, facilitating auditory perception. Research shows that the primary cause of hearing loss is the involuntary regeneration of damaged hair cells in the mammalian inner ear [9]. The investigation into the auditory impairment mechanism hinges upon the intricate functioning of the Corti organ. Advances in employing specialized immunochemical techniques have significantly enhanced our comprehension of its fundamental operating principles (Figure 1).

Hair cells exhibit a unique form of planar cell polarity, with their stereocilia bundles oriented toward the peripheral region of the cochlear duct, characterized by a distinct alignment [10]. In this process, several proteins have to be targeted to specific cellular locations to signal the directionality in the cells and to build the specific polarity structure that is unique for the maintenance of the mechanotransduction apparatuses of the sensory hair cells [11–13]. Immunohistochemical techniques are employed to discern hair cell types and precisely locate native proteins contributing to cellular architecture and mechanotransduction capabilities. Furthermore, confocal microscopy enables precise visualization of the ultrastructural features and properties of cochlear lesions, thereby facilitating comprehension of auditory physiological and pathological dynamics. This innovative experimental methodology is extensively employed in fundamental research on auditory sensory cells within the cochlea. We hereby propose a comprehensive protocol designed for the meticulous examination of the mouse auditory organ, encompassing its anatomical structure, immunohistochemical staining procedures, and advanced confocal imaging techniques.

## Materials and reagents

**Biological materials**

1. Rab11a conditional knock-out alleles, Vangl2-Looptail mice (The Jackson Laboratory, catalog number: 000220)

**Reagents**

1. Phosphate-buffered saline (PBS) (GENOM, catalog number: GNM20012-5)

2. Paraformaldehyde (PFA) (Sigma-Aldrich, catalog number: 158127)

3. Triton X-100 (Sigma, catalog number: 9002-93-1)

4. Normal donkey serum (Merck, Millipore, catalog number: S30)

5. Primary antibodies:

a. Rab11a, 1:200 (Cell Signaling Technology, catalog number: 2413)

b. γ-tubulin, 1:200 (Sigma, catalog number: T6557)

c. Arl13b, 1:1500 (Tamara Caspary, Emory University, Atlanta, GA)

d. Vangl2, 1:200 (R&D Systems, catalog number: AF4815)

e. Fz3, 1:500 (gift from Jeremy Nathans, Johns Hopkins University, Baltimore, MD)

f. LGN, 1:200 (gift from Fumio Matsuzaki, RIKEN)

g. β-Spectrin, 1:200 (BD Transduction Laboratories, catalog number: 612562)

h. MyosinVIIa, 1:200 (Proteus Bioscience Inc, catalog number: 25-6790)

i. Radixin, 1:100 (Abcam, catalog number: ab52495)

j. E-Cadherin, 1:200 (Invitrogen, catalog number: 13-1700)

6. Secondary antibodies:

a. Alexa Fluor^®^ 488 AfﬁniPure donkey anti-mouse IgG (H+L), 1:1,000 (Jackson Immuno-Research Laboratories, catalog number: 715-545-151)

b. Rhodamine Red^TM^-X AfﬁniPure donkey anti-mouse IgG (H+L), 1:1,000 (Jackson Immuno-Research Laboratories, catalog number:715-295-151)

c. Alexa Fluor^®^ 647 AfﬁniPure donkey anti-mouse IgG (H+L), 1:1,000 (Jackson Immuno-Research Laboratories, catalog number:715-605-151)

7. Phalloidin, 1:1,000 (Sigma-Aldrich, catalog number: P5282)

8. Mounting medium (Vectashield Antifade Medium, catalog number: H-1200)

9. Sodium chloride (NaCl) (Sigma-Aldrich, catalog number: S3014)

10. Potassium chloride (KCl) (Sigma-Aldrich, catalog number: P9541)

11. Sodium phosphate dibasic heptahydrate (NaH_2_PO_4_·7H_2_O) (Sigma-Aldrich, catalog number: S9390)

12. Potassium dihydrogen phosphate (KH_2_PO_4_) (Sigma-Aldrich, catalog number: P9791)

**Solutions**

1. 1× PBS (see Recipes)

2. 4% PFA (see Recipes)

**Recipes**

**1. 1× PBS**

**Table:** 

Reagent	Final concentration	Amount
Sodium chloride	n/a	8 g
Potassium chloride	n/a	200 mg
Sodium phosphate dibasic heptahydrate	n/a	1.44 g
Potassium dihydrogen phosphate	n/a	240 mg
H_2_O	n/a	1,000 mL
Total	n/a	1,000 mL

Adjust pH to 7.4 with HCl and store it at room temperature.

**2. 4% PFA**

**Table:** 

Reagent	Final concentration	Amount
Paraformaldehyde (powder)	n/a	4 g
1× PBS	n/a	100 mL
Total	4%	100 mL

Store paraformaldehyde powder at 4 °C. Heat while stirring under the chemical hood at approximately 60 °C. When the solution is transparent, let it cool down and then filter with Whatman paper to remove undissolved particles. Freeze PFA aliquots in 50 mL tubes at -20 °C. Remember to wear suitable personal protective equipment.

## Equipment

1. Dissection microscope (BT, catalog number: BTS-300)

2. Large forceps (Fine Science Tools, catalog number: 11026-15)

3. Fine forceps (Fine Science Tools, catalog number: 11252-00)

4. Very ﬁne forceps (Fine Science Tools, catalog number: 11200-14)

5. Small scissors (Fine Science Tools, catalog number: 91460-11)

6. Confocal microscope (Carl Zeiss Microscopy, model: LSM 510, AxioObserver)

7. 48-well plate (Corning, catalog number: 3548)

8. Petri dish (BD Biosciences, Falcon^®^, catalog number: 351006)

## Software and datasets

1. ImageJ software (http://rsb.info.nih.gov/ij)

## Procedure

Below, we describe the step-by-step procedure for cochlear organ dissection, immunostaining, and confocal imaging in mice from E18.5 to P2.

**A. Cochlear organ dissection**

**Figure 2.**
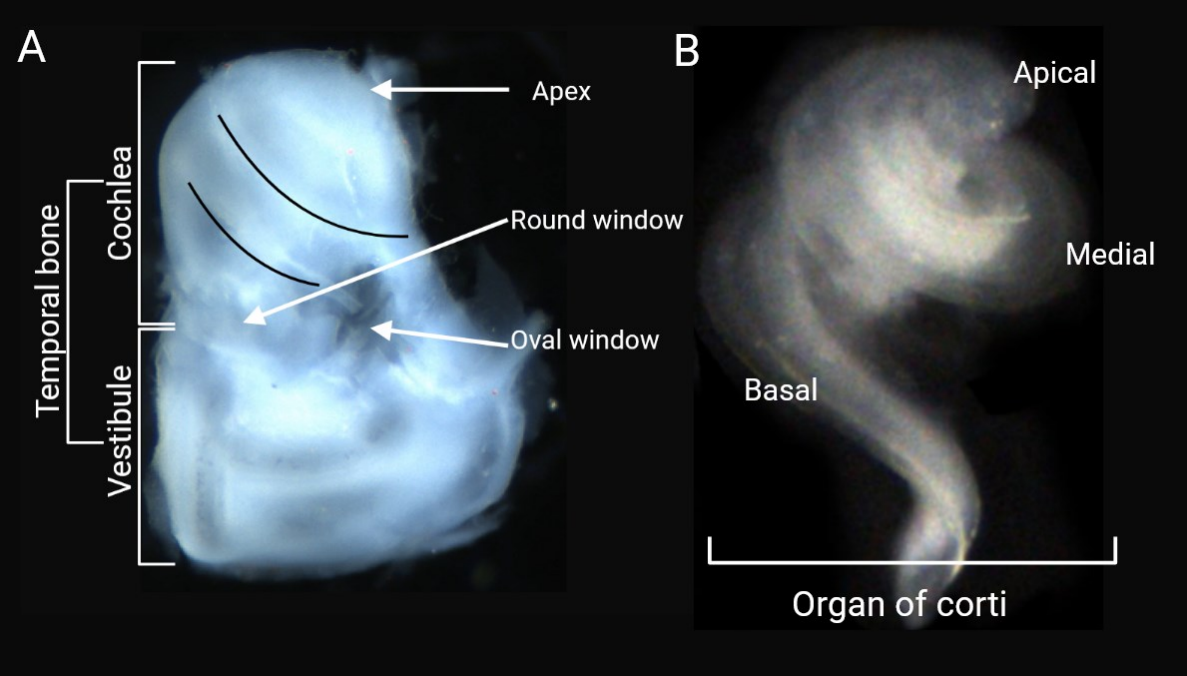
Cochlear organ dissection. A) After the removal of additional tissue from the temporal bone, the otic vesicles are revealed under a light microscope. B) Dissected organ of Corti.

1. Euthanize the mouse via cervical dislocation or CO_2_ inhalation (100% CO_2_).

2. Decapitate the mouse with small scissors. Using large forceps, remove the brain on each side to identify the temporal bones.

3. Under a dissecting microscope, use fine forceps to isolate the bony labyrinths from the temporal bones.

4. Trim away additional tissue and remove the stapes from the oval window.

5. Use the tip of the fine forceps to clear the oval and round windows and make a small hole in the apex of the cochlear spiral. Fix the bony labyrinths in 1.5 mL of 4% PFA for 5 min on ice.

6. Place the bony labyrinths in a dissecting dish containing PBS to facilitate the next step (Figure 2A).

7. Use ﬁne forceps to remove the outer cartilage to expose the cochlear duct.

8. Use ﬁne forceps to slowly remove the stria vascularis. Remove the whole stria in one attempt as far as possible.

9. Use very ﬁne forceps to remove the Reissner’s membrane. Visualize the dorsal aspect of the cochlear duct, including the sensory epithelium.

10. Use very ﬁne forceps to remove the tectorial membrane.

11. Use very ﬁne forceps to remove the intact organ of Corti (Figure 2B, Video 1).

**Video 1.**
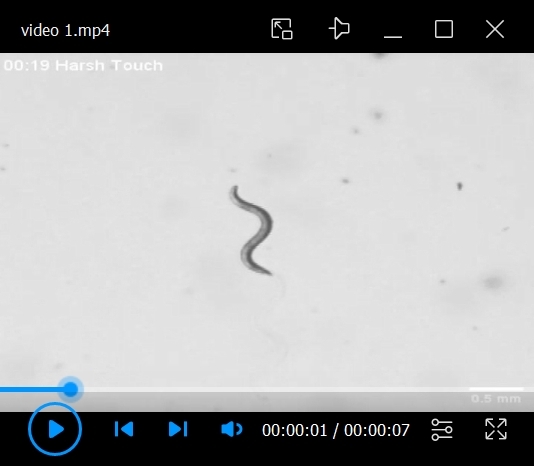
Fine dissection of the organ of Corti

12. Fix the organ of Corti immediately in the 48-well plate containing 0.5 mL of 4% PFA for 2 h at room temperature (RT), 2 h on ice, or overnight at 4 °C.

**B. Immunostaining**

1. Transfer the organ of Corti to a different well in the 48-well plate containing 0.5 mL of 0.1% Triton X-100 in PBS (PBS-T) for 10–20 min at RT. Agitation is not required for any of these steps. Transfer the organ of Corti with very fine forceps.

2. Place the tissues in 10% normal donkey serum and PBS-T for 1 h at RT.

3. Incubate the organ of Corti tissues in primary antibody in 5% normal donkey serum and PBS-T overnight at 4 °C.

4. Wash with 0.5 mL of PBS-T three times, 1–2 h each, at RT.

5. Incubate the samples in secondary antibodies in 5% normal donkey serum and PBS-T for 2 h at RT or overnight at 4 °C. This procedure should be shielded from light.

6. Wash the sample with PBS-T three times every 1–2 h at RT.

7. Incubate the tissue in phalloidin in 5% normal donkey serum and PBS-T for 30 min at RT.

8. Wash the sample with PBS-T three times every 1–2 h at RT.

**C. Confocal imaging**

1. Gently transfer the basilar membrane into a drop of PBS placed on a microscope slide.

2. Under the microscope, use very fine forceps to orient the sample facing upward toward the coverslip.

3. Add a drop of mounting medium directly onto the basilar membrane sample. Place one side of the coverslip at an angle against the slide, making contact with the outer edge of the liquid drop, and then lower the cover gently. Take care to avoid air bubbles.

4. Use an adsorbent tissue or a piece of ﬁlter paper to remove any excess mounting medium from the edges of the coverslip.

5. Seal the coverslip to the glass slide with nail polish. The sample should be stored at 4 °C in a dark environment until observation.

6. Check the intensity of the labeling and evaluate the background of the experiment by looking at the negative controls.

7. Acquire all pictures in the same conditions (exposure and general settings) for each color channel.

8. Visualize with Zeiss LSM510 confocal microscope equipped with the ZEN software using 20–63× objectives (oil immersion, NA 1.4) and the 488- and 555-nm excitation wavelengths of the laser.

9. Use the Z projection function to provide a two-dimensional view of all the pictures of an image stack by projecting them along the axis perpendicular to the image plane. The *Maximum Intensity* option creates an image in which each pixel contains the maximum value over all images in the stack.

10. Deﬁne speciﬁc positions along the cochlear duct, such as 25%, 50%, and 75% from the base, and use these to compare the proteins of interest in cochlear regions.

11. Take pictures using the microscope software for quantiﬁcation of the particular phenotype as required.

a. Stereocilia morphology: Measure the bundle convexity or the height, defined as the shortest distance between the vertex of the bundle and the line that connects two ends of the bundle. For each genotype, three independent cochleae were counted and quantified, noting the region and hair cell type.

b. Stereocilia orientation: Assess the orientation of each individual bundle relative to a line perpendicular to the planar axis. The angle is defined between the center line and the horizontal line. One line is drawn to bisect the middle of the V-shaped stereocilia bundle, and another line is drawn perpendicular to the planar axis.

c. Cilia quantification: Cilia marked by Arl13b immunostaining are recorded and counted according to different IHC rows and OHC rows. A minimum of 300 IHCs and 1,200 OHCs are counted per region of each genotype. Five individual animal samples are included in the quantification.

**Figure 3.**
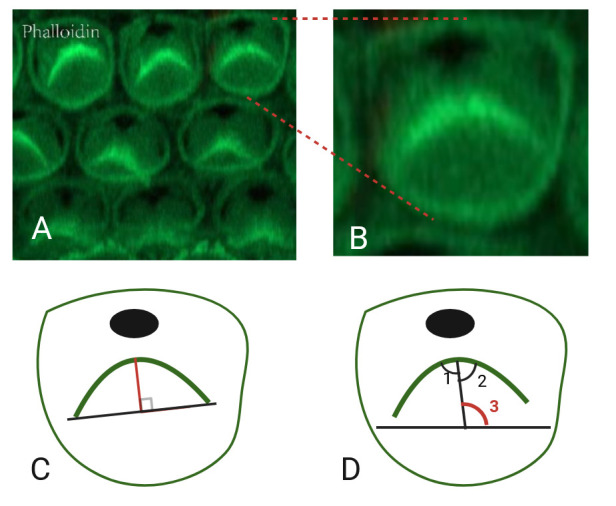
Cochlear hair cells and quantiﬁcation of the particular aspects. A) Phalloidin staining (green) in the cochlea, showing the stereocilia bundles of hair cells. B) Zoomed-in view of a single hair cell. C) Schematic diagram of length measurement. The red line indicates the length. D) Schematic diagram of angle measurement. One line indicates the bisector of the angle in V-shaped stereocilia bundles (angle 1 and angle 2 are equal). The other line indicates the horizontal line (angle 3, marked in red, indicates the measurement angle).

12. The above procedures are completed using ImageJ software. The specific steps of the software are as follows (Figure 3):

a. Open the images in ImageJ.

b. Use the straight line tool from the toolbar to connect two ends of the bundle.

c. Use the straight line tool from the toolbar to measure the shortest distance between the vertex of the bundle and the line.

d. Click *Analyze* > *Measure* and the length value will appear.

e. Use the angle tool from the toolbar to measure the V-shaped stereocilia bundle.

f. Use the straight line tool from the toolbar to bisect the middle of the V-shaped stereocilia bundle (angle 1 and angle 2 are equal).

g. Use the angle tool from the toolbar to measure the angle between the bisect line and the horizontal line (angle 3).

h. Click *Analyze* > *Measure* and the angle value will appear.

## Validation of protocol

This protocol or parts of it has been used and validated in the following research article:

- Knapp et al. [14]. Rab11a Is Essential for the Development and Integrity of the Stereocilia and Kinocilia in the Mammalian Organ of Corti. *eNeuro* (Figure 1, panels A–D; Figure 4, panels A–E; Figure 5, panels A–H; Figure 6, panels A and B; Figure 7, panels F–I).

## General notes and troubleshooting

**Troubleshooting**

Problem: Unsatisfactory immunostaining results.

Possible cause: Poor quality of samples collected; poor quality of antibodies used; irregular staining procedures.

Solution: Fresh samples should be used as much as possible to prevent problems such as degradation of proteins and severe dehydration. Ensure that the antibody is within its expiration date and use the same batch of antibody as much as possible. Note that it is necessary to avoid light when staining the secondary antibody.

## References

[1] Jang M. W., Lim J., Park M. G., Lee J. and Lee C. J. (2022). Active role of glia‐like supporting cells in the organ of Corti: Membrane proteins and their roles in hearing. Glia. 70(10): 1799-1825. 35713516 10.1002/glia.24229

[2] Bieniussa L., Jain I., Bosch Grau M., Juergens L., Hagen R., Janke C. and Rak K. (2023). Microtubule and auditory function– an underestimated connection. Semin Cell Dev Biol. 137: 74-86. 35144861 10.1016/j.semcdb.2022.02.004

[3] Xia A., Udagawa T., Quiñones P. M., Atkinson P. J., Applegate B. E., Cheng A. G. and Oghalai J. S. (2022). The impact of targeted ablation of one row of outer hair cells and Deiters’ cells on cochlear amplification. J Neurophysiol. 128(5): 1365-1373. 36259670 10.1152/jn.00501.2021 PMC9678430

[4] Buswinka C. J., Rosenberg D. B., Simikyan R. G., Osgood R. T., Fernandez K., Nitta H., Hayashi Y., Liberman L. W., Nguyen E., Yildiz E., .(2024). Large-scale annotated dataset for cochlear hair cell detection and classification. Sci Data. 11(1): 416. 38653806 10.1038/s41597-024-03218-y PMC11039649

[5] Prajapati-DiNubila M., Benito-Gonzalez A., Golden E. J., Zhang S. and Doetzlhofer A. (2019). A counter gradient of Activin A and follistatin instructs the timing of hair cell differentiation in the murine cochlea. eLife. 8: e47613. 31187730 10.7554/eLife.47613 PMC6561706

[6] Wan L., Lovett M., Warchol M. E. and Stone J. S. (2020). Vascular endothelial growth factor is required for regeneration of auditory hair cells in the avian inner ear. Hear Res. 385: 107839. 31760261 10.1016/j.heares.2019.107839

[7] Liu Y., Qi J., Chen X., Tang M., Chu C., Zhu W., Li H., Tian C., Yang G., Zhong C., .(2019). Critical role of spectrin in hearing development and deafness. Sci Adv. 5(4): eaav7803. 31001589 10.1126/sciadv.aav7803 PMC6469942

[8] Dong S. H., Kim S. S., Kim S. H. and Yeo S. G. (2020). Expression of aquaporins in inner ear disease. Laryngoscope. 130(6): 1532-1539. 31593306 10.1002/lary.28334

[9] McQuate A., Knecht S. and Raible D. W. (2023). Activity regulates a cell type-specific mitochondrial phenotype in zebrafish lateral line hair cells. eLife. 12: e80468. 36912880 10.7554/eLife.80468 PMC10129330

[10] Scheffer D. I., Zhang D. S., Shen J., Indzhykulian A., Karavitaki K. D., Xu Y. J., Wang Q., Lin J. C., Chen Z. Y., Corey D. P., .(2015). XIRP2, an Actin-Binding Protein Essential for Inner Ear Hair-Cell Stereocilia. Cell Rep. 10(11): 1811-1818. 25772365 10.1016/j.celrep.2015.02.042 PMC4376604

[11] Yu D., Deng D., Chen B., Sun H., Lyu J., Zhao Y., Chen P., Wu H. and Ren D. (2022). Rack1 regulates cellular patterning and polarity in the mouse cochlea. Exp Cell Res. 421(2): 113387. 36252648 10.1016/j.yexcr.2022.113387

[12] Okamoto S., Chaya T., Omori Y., Kuwahara R., Kubo S., Sakaguchi H. and Furukawa T. (2017). Ick Ciliary Kinase Is Essential for Planar Cell Polarity Formation in Inner Ear Hair Cells and Hearing Function. J Neurosci. 37(8): 2073-2085. 28115485 10.1523/JNEUROSCI.3067-16.2017 PMC6705687

[13] Krey J. F., Chatterjee P., Dumont R. A., M. O’Sullivan, Choi D., Bird J. E. and Barr-Gillespie P. G. (2020). Mechanotransduction-Dependent Control of Stereocilia Dimensions and Row Identity in Inner Hair Cells. Curr Biol. 30(3): 442 454 454 .e7. 31902726 10.1016/j.cub.2019.11.076 PMC7002276

[14] Knapp L., Sun H., Wang Y. M., Chen B. J., Lin X., Gao N., Chen P. and Ren D. (2023). Rab11a Is Essential for the Development and Integrity of the Stereocilia and Kinocilia in the Mammalian Organ of Corti. eNeuro. 10(6): ENEURO.0420–22.2023. 10.1523/ENEURO.0420-22.2023 PMC10249950 37225424

